# MMP-1 is a (pre-)invasive factor in Barrett-associated esophageal adenocarcinomas and is associated with positive lymph node status

**DOI:** 10.1186/1479-5876-8-99

**Published:** 2010-10-14

**Authors:** Martin Grimm, Maria Lazariotou, Stefan Kircher, Luisa Stuermer, Christoph Reiber, Andreas Höfelmayr, Stefan Gattenlöhner, Christoph Otto, Christoph T Germer, Burkhard HA von Rahden

**Affiliations:** 1 Department of General-, Visceral-, Vascular and Pediatric Surgery, University of Wuerzburg Hospital, Oberduerrbacher Strasse 6, 97080 Wuerzburg, Germany; 2 Department of Cardiac and Thoracic Surgery, University of Wuerzburg Hospital, Oberduerrbacher Strasse 6, 97080 Wuerzburg, Germany; 3 Institute of Pathology, University of Wuerzburg, Josef-Schneider Strasse 2, 97080 Wuerzburg, Germany; 4 Institute of Pathology, Medical University of Graz, Auenbruggerplatz 25, 8036 Graz, Austria

## Abstract

**Background:**

Esophageal adenocarcinomas (EACs) arise due to gastroesophageal reflux, with Barrett's esophagus (BE) regarded as precancerous lesion. Matrix metalloproteinases (MMPs) might play a role during the multistep carcinogenetic process.

**Methods:**

Expression of MMP-1 and -13 was analyzed in esophageal cancer (n = 41 EAC with BE, n = 19 EAC without BE, and n = 10 esophageal squamous-cell carcinomas, ESCC), furthermore in BE without intraepithelial neoplasia (IN) (n = 18), and the cell line OE-33. MMP-1 was co-labelled with Ki-67 (proliferation), Cdx-2 (marker for intestinal metaplasia, BE) and analyzed on mRNA level. MMP-1 staining results were correlated with clinicopatholocical parameters.

**Results:**

On protein level, MMP-1 expression was found in 39 of 41 (95%) EAC with BE, in 19 of 19 (100%) EAC without BE, in 6 of 10 (60%) ESCC, and in 10 of 18 (56%) BE without IN. No expression of MMP-13 was found in these specimens. Quantification showed 48% MMP-1 positive cells in EAC with BE, compared to 35% in adjacent BE (p < 0.05), 44% in EAC without BE, 32% in ESCC, and 4% in BE without IN. Immunofluorescence double staining experiments revealed increased MMP-1 expressing in proliferating cells (MMP-1+/Ki-67+) (r = 0.943 for BE and r = 0.811 for EAC). On mRNA-level, expression of MMP-1 was significantly higher in EAC compared to BE (p = 0.01) and confirmed immunohistochemical staining results. High MMP-1 levels were associated with lymph node metastases but not with poorer survival (p = 0.307).

**Conclusions:**

Our findings suggest that MMP-1 plays a role as preinvasive factor in BE-associated EAC. Expression of MMP-1 in proliferating BE and EAC cells suggest malignant proliferation following the clonal expansion model.

## Background

### Esophageal Adenocarcinomas and Barrett's Esophagus

Esophageal adenocarcinoma is an entity of increasing clinical importance, due to an unexplained incidence rise among white males in the Western world [1], and a dismal prognosis [2,3]. Chances for cure are still limited to early, surgically resectable tumor stages, prior to systemic dissemination of the disease. Esophageal adenocarcinomas develop almost exclusively in the distal third of the esophagus, under the chronically damaging effect of gastroesophageal reflux [2,3]. Barrett's esophagus - defined as columnar lined epithelium in the distal esophagus, characterized by specialized intestinal mucosa (with goblet cells) - is regarded as precancerous lesion, giving rise to these tumors.

Malignant progression within Barrett's esophagus (BE) is regarded to follow a sequence of well-characterized histopathologic changes, from intestinal metaplasia, over low-grade and high-grade intraepithelial neoplasia towards invasive esophageal adenocarcinomas (EAC) [2,3]. However, not all EACs are associated with BE in surgical series [4,5], and only a minority of patients with Barrett's esophagus finally progress to cancer, with an incidence between 0.5 and 2.0% per year [6].

These and other findings have raised doubt about the relevance of Barrett's esophagus as the precancerous lesion of EAC (e.g. [7]), stimulating the search for the cell population, from which esophageal adenocarcinomas originate, which is currently unknown.

The cell that gives rise to Barrett metaplasia is not known. Recently, it has been hypothesized that intestinal metaplasia may arise from a change in the differentiation pattern of *stem cells *that either reside in the esophagus or are recruited via the hematogenous route from the bone marrow [8]. In addition, due to the multistep carcinogenesis, the *clonal selection model *implies that malignant transformation occurs by multiple mutations in a random single cell and subsequent clonal selection takes place [9-11].

Evidence is accumulating, that matrix metalloproteinases (MMPs) may drive carcinogenesis according to a model of multistep carcinogenesis or a cancer stem cell hypothesis mediated by the integrin collagen receptor alpha(2)beta(1)-integrin pathway, which may also apply to esophageal adenocarcinomas [11-15]. MMPs are a family of highly homologous protein-degrading zinc dependent enzymes, functioning as endopeptidases. This family currently includes more than 25 members that can be divided into collagenases (MMP-1, -8, and -13), gelatinases (MMP-2 and 9), stromelysins (MMP-3 and 10), matrilysins (MMP-7 and 26), and the membrane-type MMPs (MMP-14 to 17 and 24). Furthermore MMPs are able to degrade the basement membrane of vessels which is essential for tumor invasion into blood and lymph vessels [14,16,17].

MMP-1 is a fibroblast-type or interstitial collagenase and majorly secreted from fibroblasts, keratinocytes, macrophages, but also cancer cells. MMP-13 is another tumor-derived MMP that is implicated to have cooperative effects with MMP-1 and is related to cancer aggressiveness [18]. No data are currently available which connect expression of MMP-1 and MMP-13 with Barrett's metaplasia and related EACs with the tumor proliferation model of multistep carcinogenesis and clinicopathologic features. The aim of our study was to investigate expression of collagenases MMP-1 and -13 in EAC (with and without associated BE) as well as non-dysplastic BE (without evidence of intraepithelial neoplasia and carcinoma) and ESCC. We aimed to indicate their potential role as preinvasive factors in BE, to compare expression levels with adjacent EACs, and to investigate a potential impact of MMP expression on survival, as well as correlation with clinicopathologic features.

## Methods

### Patients and Tumor Specimen

Surgical specimen from altogether 70 patients, having undergone primary surgical resection for esophageal cancer between January 2001 and June 2004 were included in our study, furthermore n = 18 biopsies from patients with non-dysplastic BE (without evidence of invasive carcinoma or intraepithelial neoplasia). Patients having undergone preoperative antineoplastic therapies (chemoradiation/chemotherapy) were excluded. Only patients in whom complete (R0) resection had been achieved were included.

We used archieval formalin-fixed, paraffin-embedded tissue from routine histopathologic work-up, which had been performed under standardized conditions. The material had been stored with permission of the local ethics committee, after informed consent obtained from the patients prior to surgical resection.

There were n = 41 esophageal adenocarcinomas (EAC) with associated Barrett's esophagus (BE), n = 19 EAC without BE and n = 10 esophageal squamous-cell carcinomas of the esophagus (which were intended to serve as positive control for MMP-1 expression), and n = 18 Barrett's biopsies without intraepithelial neoplasia or invasive carcinoma which were derived from patients with gastroesophageal reflux disease (GERD). EAC without BE was defined based on clinical information (endoscopic evidence of Barrett's mucosa), through work-up of all tumor blocks (searching for specialized intestinal metaplasia) and Cdx-2 staining performed in addition, which has a 70% sensitivity for staining intestinal metapasia [19].

Of note, the 19 EAC without BE were tumors in the distal esophagus (AEG type I tumors, according to the classification by Siewert and Stein, 1998, Br J Surg [20]), and explicitly not localized at the level of the anatomic gastric cardia (AEG type II tumors). The AEG type II adenocarcinoma is a tumor entity on its own and must be discussed differently.

Follow-up data were obtained from our local tumor registry of Lower Franconia/Germany. This tumor registry documents all cancer patients in the area of Lower Franconia/Germany. Information were obtained according to clinical visits of the patients (after 6 months, 12 months, 18 months, and thereafter one clinical visit per year). Information about patients who did not participate in follow-up investigations were obtained from general practitioners. Follow-up was complete for all patients (100%). Mean follow-up accounted for (29 months ± 17.6 standard deviation). Patient and tumor characteristics are given in Table 1.

**Table 1:** Clinicopathological characteristics of the EAC study population (with and without histological proven BE)

Characteristics	Patients (n = 60)	MMP-1 expression EAC		p-value
		Low (<46%)	High (≥ 46%)	
Age (y)				.605
<66	30 (50%)	13 (43%)	17 (57%)	
≥66	30 (50%)	14 (47%)	16 (53%)	
Gender				.465
Male	52 (87%)	24 (46%)	28 (54%)	
Female	8 (13%)	5 (63%)	3 (37%)	
Histological classification				.032^†^
G1	17 (28%)	11 (65%)	6 (35%)	
G2	22 (37%)	12 (55%)	10 (45%)	
G3/4	21 (33%)	6 (29%)	15 (71%)	
Depth of invasion				.163^††^
pT1	16 (27%)	9 (56%)	7 (44%)	
pT2	26 (43%)	14 (54%)	12 (46%)	
pT3	10 (17%)	3 (30%)	7 (70%)	
pT4	8 (13%)	3 (38%)	5 (62%)	
Lymph nodes metastasis				.016
pN0	23 (38%)	16 (70%)	7 (30%)	
pN1-3	37 (62%)	13 (35%)	24 (65%)	
UICC stage				.163^†††^
UICC I	14 (23%)	8 (57%)	6 (43%)	
UICC II	28 (47%)	15 (54%)	13 (46%)	
UICC III	18 (30%)	6 (33%)	12 (67%)	
UICC IV	0 (0%)	0 (0%)	0 (0%)	
Median OS (m)	43 m	47 (n = 29)	38 (n = 31)	

EAC, esophageal adenocarcinomas; BE, Barrett metaplasia; y, years; G, grading; UICC, International Union against Cancer; R, residual tumor; OS, overall survival; m, months.

^†^G1/2 vs. GT3/4; ^††^pT1/2 vs. pT3/4; ^†††^UICC I/II vs. UICC III/IV

### Histopathologic Analysis, Tumor Staging and Definition of Barrett's mucosa

Tumor blocks of paraffin-embedded tissue were selected by two experienced gastrointestinal pathologists (Stefan Kircher, Stefan Gattenlöhner) on routine hematoxylin and eosin (H&E) stained sections. Sections from all available tumor blocks of all cases underwent intensive histopathologic assessment, blinded to the prior histopathology report. H&E stained sections were analyzed with special focus on tumor infiltrated areas (EAC/ESCC), stromal areas and infiltrating immune cells. Tumor staging was performed according to the 6^th ^edition of the TNM staging system by the UICC/AJCC of 2002 [21]. Grading was performed according to WHO criteria [22].

Tumor characteristics (UICC stage, pT-categories, pN-categories, cM-categories, number of removed lymph nodes, number of tumor infiltrated lymph nodes, residual tumor status, location) and patient characteristics were documented in a database (EXCEL, Microsoft).

Barrett's mucosa was defined as specialized intestinal metaplasia (IM), characterized by goblet cells and disturbed glandular architecture [2,3]. In addition, immunohistochemistry with caudal type homeobox transcription factor 2 (Cdx-2), which is suggested as early marker for intestinal metaplasia [23], was used to identify tiny foci of intestinal metaplasia.

Furthermore, different degrees of high-grade and low-grade intraepithelial neoplasia within Barrett's mucosa were assessed. EACs were classified as "EAC with BE", when at least tiny foci of intestinal metaplasia were found due to Cdx-2 staining. EAC were classified as "EAC without BE", when the pathologists could not find intestinal metaplasia on any of the tumor blocks.

### Immunohistochemical and immunofluorescent staining

Unconjugated MMP-1, Ki-67, and isotype control antibodies were purchased from Acris (Hiddenhausen, Germany). The unconjugated Cdx-2 antibody was obtained from Biogenex (San Ramon, USA) and the unconjugated MMP-13 was provided by NeoMarkers (Asbach, Germany). The secondary antibody used for immunofluorescence double staining of Ki-67 was a fluoresceinisothiocyanat (FITC)-conjugated AffiniPure donkey-anti-rabbit IgG, used at 1:200 dilution (Jackson ImmunoResearch Laboratories Inc., Suffolk, England). The secondary antibody for MMP-1 was a Cy3-conjugated AffiniPure donkey-anti-mouse IgG (Jackson ImmunoResearch), used at 1:200 dilution.

### Cell Culture

We analyzed MMP-1 and MMP-13 expression in cells (1 × 10^4^) from the esophageal adenocarcinoma cell line OE-33 (Sigma-Aldrich, Steinheim, Germany) in cytospins. This cell line is the only commercially available adenocarcinoma cell line of the lower esophagus (Barrett's metaplasia) and was established from a 73-year-old female patient. The tumor was identified as pathological stage IIA (UICC) and showed poor differentiation. Using RT-PCR we tested negative for mycoplasma contamination of this cell line that was provided to our laboratory in December 2009 by Sigma. The cell line was cultured in RPMI-1640 medium, supplemented with 10% Fetal Bovine Serum, 100 units/ml of penicillin and 100 μg/ml of streptomycin. Cytospins of the OE-33 cell line were fixed in acetone and dried for 10 minutes. Rehydration, blocking, and the staining procedure steps were the same as described for immunohistochemistry of FFPE sections. Additionally, RT-PCR was performed for MMP-1 gene expression of OE-33 cells.

### Double Staining Experiments (IF and IHC)

The sequential immunofluorescence (IF) double staining (co-expression) was analyzed for MMP-1 with Ki-67 expression. Sequential immunohistochemical (IHC) double staining was performed for Cdx-2 and MMP-1.

### Processing of tissue and staining procedure

First we assessed H&E sections from each tumor tissue to differentiate between BE, tumor cell areas, stromal areas and infiltrating immune cells. We then stained for MMP-1, -13, Cdx-2, and Ki-67 in additional serial sections of 2 μm thickness. Tissue sections (2 μm thickness) were cut from paraffin blocks on a microtome and mounted on adhesive microscope slides (Hartenstein, Wuerzburg, Germany). Serial sections were deparaffinized in xylene and ethanol and rehydrated in water. Heat induced epitope retrieval (HIER) was performed with citrate buffer pH 6.0 (Dako, Hamburg, Germany). For IF, slides were then incubated in normal serum (2%) and bovine serum albumin (BSA) (0.5%) at room temperature for 20 minutes to block nonspecific binding. Subsequently, slides were incubated with the primary antibody or control antibody overnight at 4°C in a humidified chamber and with secondary FITC-conjugated antibody for 30 minutes at room temperature in a humidified chamber. The slides were incubated with the second primary antibody diluted in TBS plus 0.5% BSA overnight at 4°C, followed by incubation with the secondary Cy3-conjugated antibody for 30 minutes at room temperature. Slides were counterstained with DAPI (4',6-Diamidino-2-phenylindoldihydrochlorid, Sigma-Aldrich) and covered with Polyvinyl-alcohol mounting medium (DABCO, Sigma-Aldrich) and analyzed using a Zeiss camera (Jena, Germany). The photographed images using the Metamorph software package (Visitron Systems, Puchheim, Germany) were imported into the Microsoft Office Picture Manager.

For IHC, the pretreatment procedure (fixation, deparaffinization, rehydration, HIER, and blocking) of the slides was the same as described for IF. For immunohistochemical analysis a four-step immunoperoxidase labeling for single antigens in formalin-fixed, paraffin-embedded sections was used as described [24]. For immunohistochemical double staining, we first used an alkaline phosphatase (AP)-conjugated AffiniPure Donkey anti-mouse Ab followed by 20 minutes of incubation with Fast Red (Dako). After incubation with the second primary antibody, we used a horseradish peroxidase (HRP)-conjugated AffiniPure Donkey anti-rabbit IgG (Jackson ImmunoResearch) followed by 5 minutes of incubation with DAB (Biogenex).

### Quantification of Immunohistochemistry (IHC) and Immunofluorescence (IF)

MMP-1 and Ki-67 IHC was quantified in EAC with BE, as well as in the associated Barrett's mucosa, as well as EAC without BE. Quantification of immunoenzymatic staining of IN or tumor cells was performed, analyzing six representative individual high power fields (×400) for each sample. Scoring was done by means of cell counting. The results were expressed as percentages (number of positive cells within 100 counted tumor cells, %). Sections were evaluated by two independent blinded investigators separately. In case of discrepancies, both evaluated the slides simultaneously and made an agreement. For each tumor section, quantification of IF double staining was performed by counting Ki-67+ cells in six microscopic high power fields (400 × magnification) in parallel with MMP-1+. The proportion of Ki-67 positivity in counted MMP-1+ cells was expressed in percentages.

### Real-time quantitative reverse transcription-PCR analysis

To analyze gene expression of MMP-1 by RT-PCR in FFPE tissue, we extracted total cellular RNA and performed cDNA synthesis using the Absolutely RNA FFPE Kit and the AffinityScript QPCR cDNA Synthesis Kit from Stratagene (Waldbronn, Germany). Areas of interest for each tissue section were manually microdissected. For both groups (BE and EAC) equal amounts of tissue areas were assessed (2 × 1.5 cm^2 ^surface area per section, thickness of 10 μm). For OE-33 cell line, after homogenization Diethyl pyrocarbonate (DEPC)-75% ethanol was added to the lysate to provide ideal binding conditions. The lysate was then loaded onto the RNeasy silica membrane ("RNeasy Mini spin column", RNeasy Mini Kit Qiagen, Hilden, Germany). RNA binds, and all contaminants were washed away efficiently. Pure, concentrated RNA was eluted in water and stored at -70°C until further analysis. The amount of total RNA was determined by measuring absorbance at 260 nm. The purity of total RNA was established by confirming that the 260 nm:280 nm ratio was within a 1.8-2.0 range, indicating that the RNA preparations were free of protein contaminants. Primers were designed using the Primer Express software for primer design to amplify short segments of 50-150 base pairs of target cDNA. The MMP-1 forward primer sequence was: 5'-TGCTGCTGCTGCTGTTCTGGG-3'; the MMP-1 reverse primer sequence was: 5'-GGCCGATGGGCTGGACAGGA-3'. Matched human esophageal cDNA was purchased by BioChain (Hayward, CA, USA) as control. The housekeeping gene Glyceraldehyde-3-phosphate dehydrogenase (GAPDH) was used for relative quantification and cDNA quality control. The GAPDH forward primer sequence was: 5'-ATCCCATCACCATCTTCCAGG-3'; the GAPDH reverse primer sequence was: 5'-CGCCCCACTTGATTTTGG-3'. RT-PCR reactions were carried out as described previously [25]. The relative quantification value, fold difference, is expressed as 2^-ΔΔCt^.

### Statistical analysis

Statistical analysis was performed with MedCalc Software, Version 11.3.2 (Mariakerke, Belgium). All values were expressed as Median ± Interquartile Range (IQR) because D'Agostino-Pearson test did not show a normal distribution of gene and protein expression. Therefore, the Median value was chosen to divide patients in two different groups. Survival time was determined as the time from tumor resection to tumor conditional death and as the time from tumor resection to time of obvious recurrence. The overall survival (OS) time in association with MMP-1 expression was estimated using the Kaplan-Meier method [26]. To analyze differences in the overall/tumor related survival among patients after successful (R0) curative surgical resection for EAC patients were divided into two subgroups (dichotomous variables). Median cut-off value for either high or low expressors was set at 46% for MMP-1 expression in all EAC (n = 60). The log rank test was used to check for statistical differences between the survival curves. Cases with less than 10% positive cells were regarded as negative.

Multivariate analyses were performed using the Cox Proportional Hazards Model. All parameters that were found significant on univariate analysis were included [27].

Correlation analysis was performed by the non-parametric Spearman Rho rank correlation coefficient. Fisher's exact test was used to investigate the relation between two categorical variables. Data were analyzed using the non-parametric Mann-Whitney U test or Kruskal-Wallis test when more than 2 groups were compared. P values of less than 0.05 were regarded statistically significant.

## Results

### MMP-1 expression is associated with BE and associated EAC

MMP-1, MMP-13 and Cdx-2 were not expressed in normal esophageal squamous epithelium. MMP-1 expression in stromal cells was considerably weak and strongly associated with high-grade and low-grade intraepithelial neoplasia within Barrett's mucosa as well as cancer cells. 95% (n = 39/41) of the patients with BE and adjacent EAC expressed MMP-1 within the tumor. Similarly, 100% (n = 19/19) of the EAC without BE expressed MMP-1, 6 of 10 ESCC (60%), and 10 of 18 (56%) Barrett's biopsies without intraepithelial neoplasia or carcinoma stained positive for MMP-1.

Expression of MMP-13 was negative in tumor specimen and in the esophageal adenocarcinoma cell line OE-33, but was rarely detected in stromal cells (data not shown).

**Figure 1:**
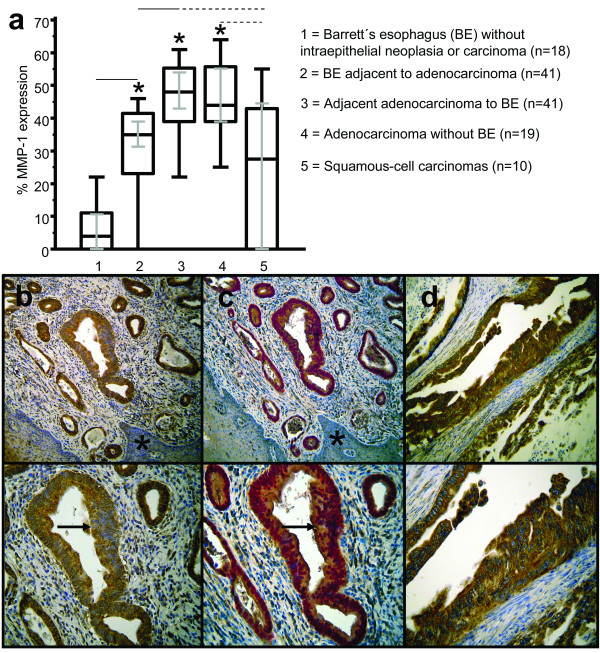
**Immunohistochemical analysis and staining of MMP-1 in human BE and EAC**. In comparison to BE without intraepithelial neoplasia (GERD) (1) a significantly (p < 0.05) increased expression of MMP-1 was observed in BE adjacent to EACs (2). Expression levels of MMP-1 were significantly (p < 0.05) increased in associated EACs (3) and EACs without BE (4). ESCC showed significantly (p < 0.05) decreased MMP-1 expression compared to EACs (5). Analysis refers to percentages of positivity of all counted cells. Grey lines show 95% confidence intervals. Statistically significant values from BE and ESCC to EACs are indicated with asterisks (**a**). Increased expression of MMP-1 (**b**) was observed in early BE (arrows). Adjacent normal tissue stained negative for MMP-1 (asterisk). Single staining of MMP-1 in BE was confirmed by immunohistochemical double staining (**c**), showing Cdx-2 (nuclear staining pattern, Fast red) and MMP-1 (cytoplasmic staining pattern, brown). Significantly increased MMP-1 expression was observed in adenocarcinomas compared to BE (**d**). Original magnification: top × 100, bottom × 200.

Furthermore, we analyzed positivity of all counted cells according to the precursor lesion and tumor entity. Compared to BE (GERD) without intraepithelial neoplasia or carcinoma (Figure 1a, Table 2), MMP-1 expression was significantly upregulated in BE (Figure 1a, Table 2) with adjacent EAC (Figure 1a, Table 2) and EAC without BE (Figure 1a, Table 2). No differences of MMP-1 expression were found between different degrees in high-grade and low-grade intraepithelial neoplasia within Barrett's mucosa. Median MMP-1 expression of all EACs (n = 60) was 46%, IQR 39.0 - 55.5%; 95% CI 43.0 - 54.0%.

**Table 2:** MMP-1 expression of the study population in different tissues

Tissue	n	Median expression (%)	IQR (%)	95% CI	p-value
BE without intraepithelial neoplasia or carcinoma (GERD)	18	4	0-11	0-10.603	
BE adjacent EAC	41	35	23.0-41.5	31.284-39.0	<0.05^†^
Adjacent EAC to BE	41	48	39.0-56.5	43.0-54.239	<0.05^††^
EAC without BE	19	44	39.0-55.8	39.0-55.218	<0.05^††^
ESCC	10	27	0-50.2	0-29.2	<0.05^†††^

BE, Barrett metaplasia; GERD, Gastro-Esophageal Reflux Disease; EAC, esophageal adenocarcinomas; ESCC, esophageal squamous-cell carcinomas; ^†^significance is related to GERD; ^††^significance is related to BE with adjacent EAC; ^†††^significance is related to EACs

ESCC showed significantly decreased MMP-1 expression (Figure 1a, Table 2), compared to EACs. For adenocarcinomas without BE, the results of MMP-1 expression were comparable with the higher expression levels of adenocarcinomas from BE. Expression levels of MMP-1 in ESCC did not differ significantly from BE with adjacent EAC but showed a decrease compared to BE (Figure 1a, Table 2).

**Figure 2:**
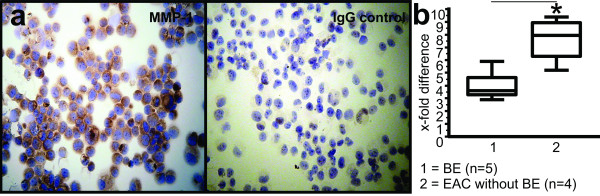
**Immunohistochemical staining of MMP-1 from the OE-33 adenocarcinoma cancer cell line and MMP-1 gene expression in human BE and EAC**. MMP-1 staining in cytospins from the OE-33 adenocarcinoma cancer cell line served as additional positive control (left) and showed 65% positive cells; IgG control (right) (**a**). Gene expression of MMP-1 in human BE and EAC. MMP-1 gene expression in BE was significantly (p = 0.01) lower in comparison to EAC without BE. Normal tissue is considered as one-fold (**b**). Statistically significant value is indicated with an asterisk.

Figure 1b demonstrates a representative example of MMP-1 expression in early BE. We confirmed areas analyzed for MMP-1 expression of BE by immunohistochemical double staining with Cdx-2 (Figure 1c). Figure 1d demonstrates a representative example of MMP-1 expression in EAC. Stainings from the OE-33 adenocarcinoma cancer cell line in cytospins served as additional positive control for MMP-1 expression and showed 65% positive cells (Figure 2a).

### Analysis of MMP-1 gene expression

To confirm the results of the immunohistochemical staining, gene expression of MMP-1 in human BE and EAC were assessed. MMP-1 gene expression in BE (Median 3.6-fold difference compared to normal tissue; IQR 3.275 to 4.625-fold difference; n = 5) was significantly (p = 0.01) lower in comparison to EAC without BE (Median 7.9-fold difference compared to normal tissue; IQR 6.3 to 8.95-fold difference; n = 4; Figure 2b). These results confirmed increased MMP-1 expression in BE and significantly elevated expression of MMP-1 in EAC without BE as observed by immunohistochemistry. RT-PCR results for MMP-1 expression in the adenocarcinoma cell line OE-33 showed a 4.1-fold higher expression compared to normal tissue.

### MMP-1 expression is strongly correlated with proliferating (Ki-67+) Barrett and EAC cells

**Figure 3:**
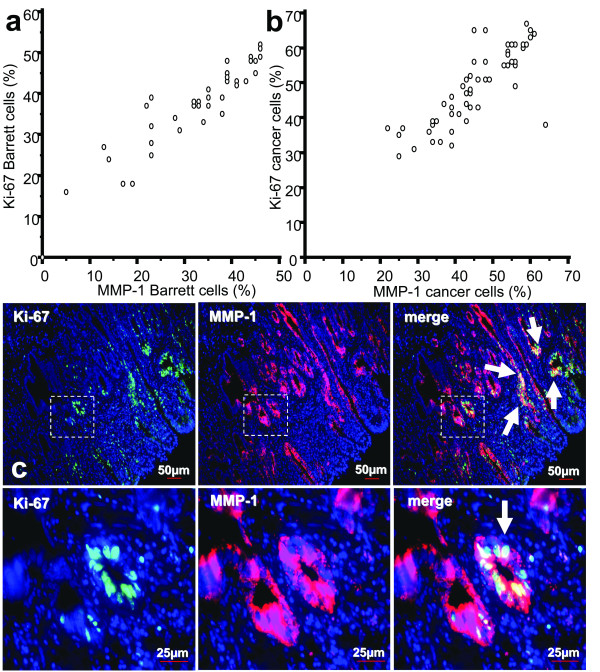
**Correlation and co-expression of Ki-67 with MMP-1**. Correlation from quantified immunohistochemical staining results of MMP-1+ in BE (n = 41) and EAC (n = 60) with proliferating cells (Ki-67+) showed that Ki-67+ expression in BE **(a) **and EACs **(b) **had a strong direct correlation with the expression of MMP-1+ (r = 0.943 for BE and r = 0.811 for EAC). **(c) **Images demonstrate a representative example of Ki-67 co-expression with MMP-1+ by an immunofluorescent double staining in early BE showing the majority of proliferating (Ki-67+) cells with MMP-1+ (big arrows). Small arrows indicate goblet cells. FITC green Fluoresceinisothiocyanat, Cy3 red, and DAPI 4',6-Diamidino-2-phenylindoldihydrochlorid blue. Top, Calibration bar represents 50 μm. Bottom, calibration bar represents 25 μm. The square box at the bottom demonstrates the area which is also shown in larger magnification.

For investigation of proliferating cells in BE and EAC and its relation to multi-step carcinogenesis, we analyzed MMP-1 expression in early Barrett cells, adjacent EAC, EAC without BE and ESCC. Evaluation of immunohistochemically stained serial sections showed a strong positive correlation of MMP-1 expression with proliferating cells (Figure 3a and 3b: MMP-1+/Ki-67+: r = 0.943 for BE, n = 41 and r = 0.811 for EAC, n = 60). As shown in Figure 3c by an immunofluorescence double staining, MMP-1 was co-expressed with great amounts of proliferating (Ki-67+) cells in areas which were associated with early BE (goblet cells as well as Cdx-2 positivity were observed in serial sections) (Figure 3c, representative example of n = 41 BE). IF double staining confirmed correlation analysis evaluated in IHC serial sections. We found a dominant population of proliferating MMP-1+/Ki-67+ cells in BE and EAC. Proliferation status (Ki-67+) itself did not have had any impact on survival (data not shown).

### Prognostic value of MMP-1 in adenocarcinomas

**Figure 4:**
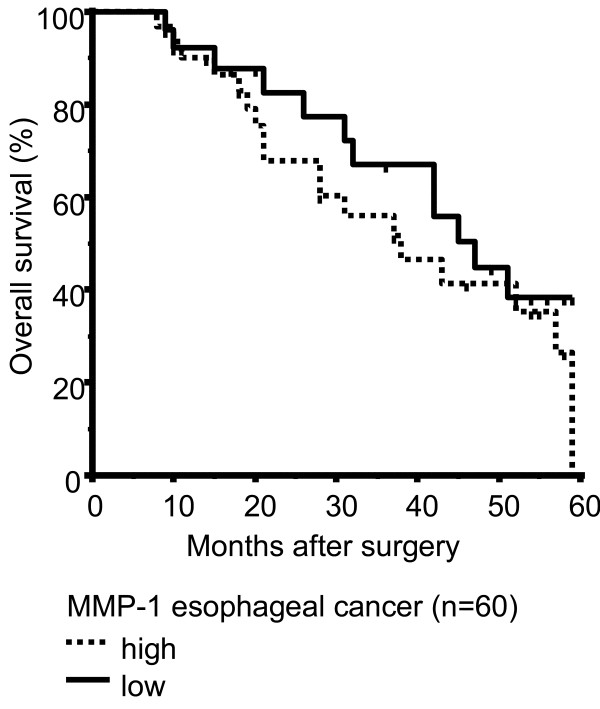
**Overall survival curves calculated by Kaplan-Meier method in Barrett associated adenocarcinomas**. High levels of MMP-1 expression in EAC (n = 60) were not found to be associated with poorer survival (p = 0.307). The times of the censored data are indicated by short vertical lines.

To analyze survival differences of patients after successful (R0) curative surgical resection for EAC with and without BE, patients were divided into two subgroups as described above (dichotomous variables). Lymph node metastasis (pN+, Table 3, pT-category (pT3/4, Table 3) and grading (G3/4, Table 3) were shown to be unfavorable factors in univariate analysis of all (n = 60) EACs. Moreover, we found a strong association between high MMP-1 expression and positive lymph node metastases (p = 0.016136582, Fisher's exact test, Table 1) in EAC patients (n = 60). To analyze differences in tumor related survival dependent on MMP-1-expression in EAC we divided the patients in two subgroups as described above (dichotomous variables). Survival in subgroup with high MMP-1 expression of all EACs (n = 60; Figure 4, Table 1) was not significantly worse in comparison to the subgroup of patients with low expression of MMP-1 (Figure 4, Table 1). Data show that MMP-1 expression in BE and adjacent EACs is associated with clinicopathologic features which may predict worse clinical outcome of adjacent EACs. Multivariate analysis using the Cox Proportional Hazards Model demonstrate lymph node metastases and grading as independent prognostic factors in all (n = 60) EACs (Table 4).

**Table 3:** Univariate analysis of prognostic factors of the patients (n = 60)

Variable	Unfavorable factor	Hazard ratio (HR)	95% CI of HR	p-value
LN	positive	12.1940	5.9509 to 24.9867	< 0.0001
Depth of invasion	pT3/4	3.8447	1.5309 to 9.6553	< 0.0001
Grading	High (G3/4)	4.0652	1.7123 to 9.6514	< 0.0001
MMP-1 expression	High (≥ 46%)	1.4526	0.7101 to 2.9718	0.3070

LN, Lymph nodes metastasis

**Table 4:** Multivariate analysis of prognostic factors of the patients (n = 60)

Variable	Unfavorable factor	Hazard ratio (HR)	95% CI of HR	p-value
LN	positive	9.1861	2.0665 to 40.8346	0.003746
Depth of invasion	pT3/4	1.2336	0.2783 to 5.4683	0.7834
Grading	High (G3/4)	2.2593	1.0171 to 5.0186	0.04643

LN, Lymph nodes metastasis

## Discussion

*'Tissue invasion and metastasis' *is one of the six 'hallmarks of cancer', initially described by Hanahan and Weinberg [28,29]. Invasive and metastatic capabilities are largely mediated by extracellular proteases, which are able to destroy the environment of the tumor cell. MMPs have been shown to be involved in this process. The early work of Liotta et al. [30] provided some of the first evidence that MMPs were involved in invasion of a tumor cell through the basement membrane. Previous studies described MMP-9 in the pathogenesis of Barrett's esophagus, ESCC, and gastric cancer [31-33]. However, we have focused on MMP-1 and MMP-13 expression, because no data have been available regarding clinicopathological factors in BE and EAC so far. Our findings of an increased MMP-1 expression in EAC is well in line with results obtained in other cancer entities and few samples of EAC without clinicopathological association [34-41] suggesting a putative role in invasion, metastasis and poorer survival. In this context, MMP-13 has been shown to play a role in tumor progression of other gastrointestinal tumor entities such as colorectal carcinoma [42], and hepatocellular carcinoma [43]. In gastric cancer and specimen with human esophageal carcinomas (ESCC) MMP-13 expression has been previously shown to contribute to malignant progression. It has furthermore been suggested that its coordinate overexpression with MMP-1 and/or MMP-2 may have a synergetic effect in tumor progression [18,44]. In sharp contrast to these findings, in our study, MMP-13 was not found expressed in EACs, but was occasionally detected in surrounding stromal cells. Therefore, based on our results, we may exclude this cooperative effect in patients with EAC.

Our findings of high MMP-1 expression being associated with lymph node metastases in patients with EAC indicate that MMP-1 expression may be involved in promotion of cancer progression in addition to other clinicopathological characteristics. However, we have not been able to demonstrate a negative impact of increased MMP-1 expression on survival.

To date molecular pathogenesis of BE is poorly understood. According to the clonal evolution model we found a dominant population of proliferating cells (Ki-67+) in EAC, which may drive multi-step carcinogenesis [45]. We have chosen to study proliferation with Ki-67, because it is a proliferation-associated nuclear antigen and expressed in all cycling cells except for resting cells in the G0 phase, which implies no negative survival effect [46] but may be associated with neoplastic progression in BE [47]. Therefore, our results may indicate that MMP-1 expression is associated with multistep carcinogenesis according to clonal selection model from BE to EAC. Furthermore, we hypothesize MMP-1 signaling in the pathogenesis of adenocarcinomas by the integrin collagen receptor alpha(2)beta(1)-integrin pathway which has been shown to be involved human prostate epithelial stem cells [15]. In osteosarcoma cells the level of cell surface alpha(2)beta(1)-integrin correlates with the expression level of native collagenase MMP-1 [48]. Therefore, the investigation of this signalling pathway might be a promising target for future investigations in the carcinogenesis of Barrett's esophagus. However, to date there are no experimental data that support this hypothesis [11].

Additionally, the findings of elevated gene and protein expression of MMP-1 by BE and EAC as preinvasive factor might also be important for esophageal squamous-cell carcinomas, although it was only investigated in a smaller sample. A critical role of MMP-1 for promoting invasion and metastasis in this tumor entity has been described earlier [49].

## Conclusions

MMP-1-expression was found in a major population of proliferating MMP-1+ Barrett and EAC cells. Expression of MMP-1 in proliferating (Ki-67+) cells of intestinal metaplasia and in Barrett-associated adenocarcinomas may thus sustain multi-step carcinogenesis and further tumor growth. These findings may contribute to the further understanding of the pathogenesis of esophageal carcinogenesis. Furthermore, MMP-1 seems to be in respect of the integrin collagen receptor alpha(2)beta(1)-pathway, which has been associated with a putative stem cells hypothesis in prostate cancer. This signaling pathway might be promising for further investigations in the carcinogenesis of Barrett-associated adenocarcinomas due to a cancer stem cell hypothesis.

## Competing interests

The authors declare that they have no competing interests.

## Authors' contributions

GM conceived the study, carried out immunohistochemistry studies, performed the statistical analyzes and drafted the manuscript. LM participated in the design of the study and performed RT-PCR studies. SK and SG participated in the design of the study, evaluated cancer samples, and helped to draft the manuscript. CR and LS participated in the design of the study, and performed immunohistochemistry studies. AH helped to draft the manuscript. CO and GCT participated in the design of the study design and coordination and drafted the manuscript. VRBHA participated in the design of the study design, performed preliminary RT-PCR and immunohistochemistry studies and drafted the manuscript. All authors read and approved the final manuscript.
